# Investigation of the role of nuclear factors associated with double-stranded RNA protein members in the pathogenesis of HTLV-2

**DOI:** 10.1186/1742-4690-11-S1-P113

**Published:** 2014-01-07

**Authors:** Jane L Murphy, William W Hall, Noreen Sheehy

**Affiliations:** 1 Centre for Research in Infectious Diseases, School of Medicine and Medical Science, University College Dublin, Ireland

## 

It has recently been demonstrated that HTLV-1 and HTLV-2 utilise antisense transcription to express the viral proteins, HBZ and APH-2, respectively. Studies have shown that HBZ is a key player in the pathogenesis of HTLV-1 as its temporal expression appears to be critical for the development of ATL and HAM/TSP. To date very little is known about the role of APH-2 in HTLV-2 infection. Thus, to investigate its role in the pathogenesis of HTLV-2, we recently performed yeast two hybrid screens of several cDNA libraries using APH-2 as bait. We identified a member of the Nuclear Factors Associated with double-stranded RNA (NFAR) proteins as a potential interacting protein of APH-2. The NFARs have been shown to be involved in the regulation of gene expression and most notably; host anti-viral defence. Our research affords the opportunity to investigate the interaction of APH-2 and NFARs and determine how this interaction impacts on HTLV-2 lifecycle events. Our findings indicate that APH-2 and NFARs interact in vivo and in vitro. Immunofluorescence demonstrates that APH-2 and NFARs localise to the nucleus. We have also performed knockdown studies to determine the effect of NFAR proteins on HTLV-2 LTR promoter activity. Our results reveal that NFARs exert no effect on basal LTR activation, but inhibit Tax2B-mediated viral transcription. Additionally we report that in the presence of APH-2, NFARs enhance Tax2B-mediated LTR activation. Overall, this study will provide novel insights into the functional role of APH-2 and NFAR proteins in the pathogenesis of HTLV-2.

